# Spatiotemporal bias of the human gaze toward hierarchical visual features during natural scene viewing

**DOI:** 10.1038/s41598-023-34829-x

**Published:** 2023-05-18

**Authors:** Kazuaki Akamatsu, Tomohiro Nishino, Yoichi Miyawaki

**Affiliations:** 1 Graduate School of Informatics and Engineering, The University of Electro-Communications, 1-5-1 Chofugaoka, Chofu, Tokyo 182-8585 Japan; 2 Faculty of Informatics and Engineering, The University of Electro-Communications, 1-5-1 Chofugaoka, Chofu, Tokyo 182-8585 Japan; 3 Center for Neuroscience and Biomedical Engineering (CNBE), The University of Electro-Communications, 1-5-1 Chofugaoka, Chofu, Tokyo 182-8585 Japan

**Keywords:** Oculomotor system, Visual system

## Abstract

The human gaze is directed at various locations from moment to moment in acquiring information necessary to recognize the external environment at the fine resolution of foveal vision. Previous studies showed that the human gaze is attracted to particular locations in the visual field at a particular time, but it remains unclear what visual features produce such spatiotemporal bias. In this study, we used a deep convolutional neural network model to extract hierarchical visual features from natural scene images and evaluated how much the human gaze is attracted to the visual features in space and time. Eye movement measurement and visual feature analysis using the deep convolutional neural network model showed that the gaze was more strongly attracted to spatial locations containing higher-order visual features than to locations containing lower-order visual features or to locations predicted by conventional saliency. Analysis of the time course of gaze attraction revealed that the bias to higher-order visual features was prominent within a short period after the beginning of observation of the natural scene images. These results demonstrate that higher-order visual features are a strong gaze attractor in both space and time, suggesting that the human visual system uses foveal vision resources to extract information from higher-order visual features with higher spatiotemporal priority.

## Introduction

Humans acquire visual information about the external world by moving their eyes to direct their gaze toward various locations. Eye movement is essential for accurate visual recognition because the spatial resolution of human vision is not uniform over eccentricity—i.e., the foveal vision possesses visual acuity higher than that of peripheral vision^1^. Given this constraint, the human visual system prioritizes particular locations by directing the gaze and processes the information using foveal vision at a high spatial resolution. However, which visual features determine such gaze biases in observed images remains under debate.

Conventionally, the spatial contrast in lower-order visual features such as luminance, color, and orientation has been considered influential in attracting the human gaze^2,3^. Classical saliency is computed as a mixture of these components. Since classical saliency is currently used in much wider definitions, we instead call the saliency of low-level features that are measured through the Itti–Koch model “the Itti–Koch saliency” in this paper. Computational models have also revealed that fixation locations can be predicted using a saliency map that quantifies a value of the Itti–Koch saliency at each spatial location in observed images^2,3^. However, the Itti–Koch saliency requires computation at not only a local scale but multiple scales including broad spatial coverage^2,3^. It is thus difficult to infer what visual cortical area is primarily involved in multiscale feature extraction processes and the generation of gaze bias. On the other hand, it may be possible to define another type of saliency based on higher-order visual features extracted in the ventral visual pathway.

Recent studies showed that activation of a higher layer of deep convolutional neural network (DCNN) models can be used to predict the gaze bias to particular locations in observed images. The prediction accuracy is much higher than that of the conventional saliency map^4–8^ (see also MIT/Tuebingen Saliency Benchmark for the list of saliency models and their evaluation^9,10^). These results suggest that higher-order visual features are more informative than lower-order visual features in predicting the fixation locations in space, possibly being assisted by the complexity of DCNN models with a large number of flexible parameters. However, in these studies, the DCNN models were combined with an additional multilayer neural network module whose parameters were tuned to predict the fixation locations, and the combination obscured whether the gaze was really attracted toward the spatial locations containing the higher-order visual features. In fact, the spatial distribution of the fixation locations was even better predicted by a combination of lower-order visual features and the multilayer neural network module than by the higher-layer activation of the DCNN model^8^. Hence, it remains unclear whether the gaze is attracted to spatial locations containing higher-order visual features.

Similar issues exist when considering which visual features attract the gaze earlier in time. Previous studies suggest that spatial locations having a higher contrast in the visual stimulus, thus having a higher intensity of the Itti–Koch saliency, attract the gaze within a short period after the stimulus onset^11,12^. On the other hand, a recent study argues that the gaze within a short period after the stimulus onset can be predicted by activation of the higher layer of DCNN models more accurately than by using a lower-order visual feature like the Itti–Koch saliency^13^. However, since Schütt et al.^13^ attached an additional neural network module to the higher layer of the DCNN model (VGG19^14^) and tuned parameters of the attached module, it remains uncertain whether the feature extraction by the hierarchical network is essential for the accurate fixation prediction.

To resolve the above issues, we examine the direct relationship between the spatiotemporal gaze bias and the visual features by mapping fixation locations and DCNN-extracted hierarchical visual features into observed images. Here, we use a DCNN model that was pretrained using an independent dataset of natural images^15^ as a hierarchical visual feature extractor without any additional modules for fixation prediction. As many studies have demonstrated, DCNN models can be considered a good model of hierarchical visual systems of the human brain, with each layer responding to visual features of different levels of complexity along its hierarchy^16–18^. This similarity allows us to infer what visual areas or levels of visual information processing in the brain affect the gaze bias in observed images. Furthermore, because the DCNN models are used as an independently pretrained filter to extract visual features at each layer in its hierarchical architecture, we can examine what and when visual features attract the gaze. In other words, this methodology is expected to reveal spatiotemporal characteristics of the gaze bias to visual features in observed images. Our approach will provide not only systematic knowledge about feature-based human gaze control but also a useful insight into effective visual designs.

## Results

We selected various images from large-scale natural scene datasets^19,20^ and used them as visual stimuli in experiments. Using these visual stimuli, we performed encoding runs in which participants observed the visual stimuli with free eye movements, followed by recognition runs in which the participants observed the visual stimuli with free eye movements and answered whether the observed stimuli were presented in the preceding encoding run. The recognition task accuracy was significantly higher than the chance level for all participants (Supplementary Fig. 2; binomial test p < 0.05 for each participant; mean ± standard deviation across participants, 91.25 ± 6.74%), indicating that all participants engaged in the experiment. We also performed a similar experiment without the recognition task (the scene images were just exposed to participants, without any task followed), but the results did not change significantly. Thus, the following results were independent of the presence or absence of the recognition task.

**Figure 1:**
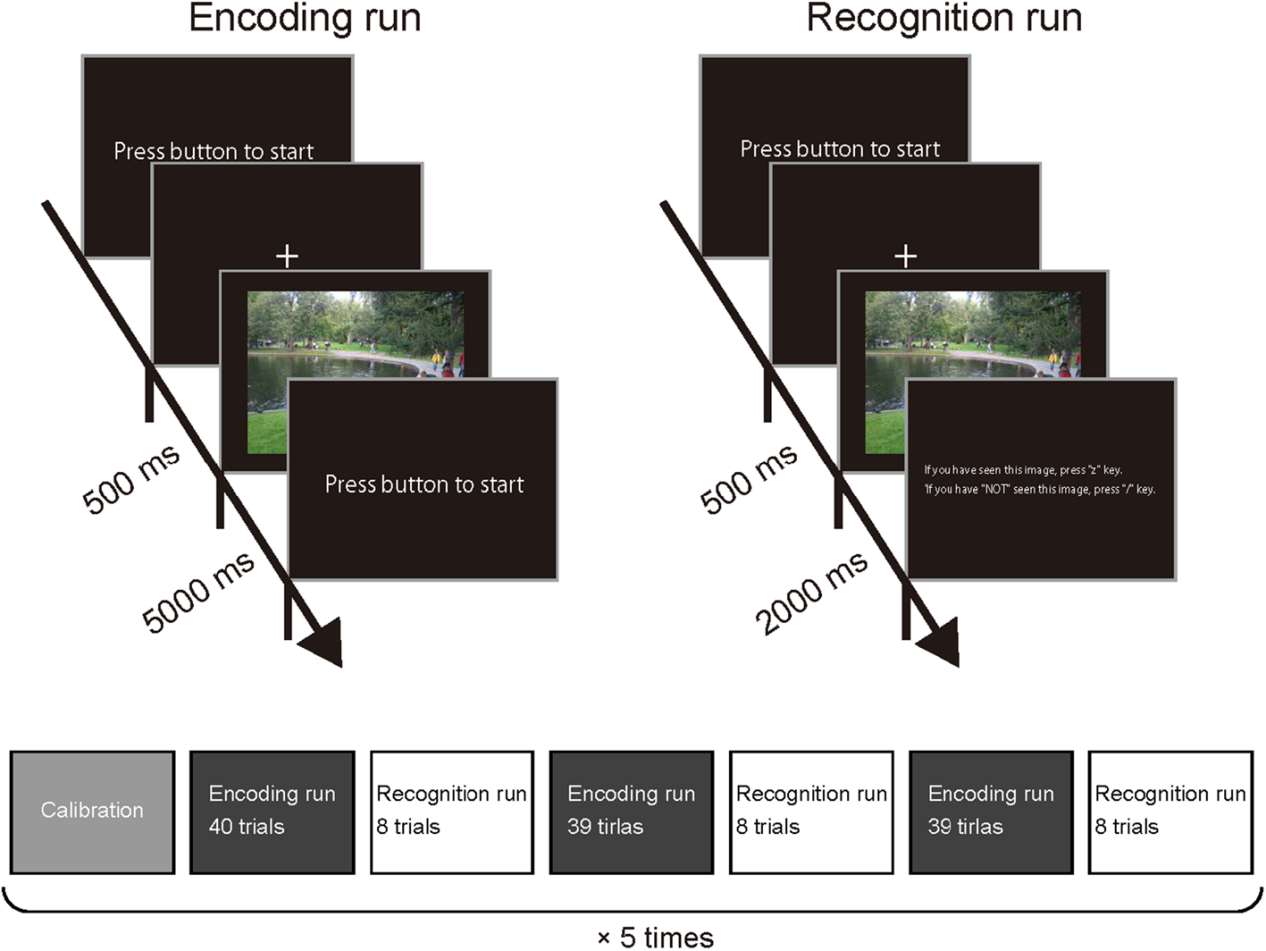
Experimental design. The experiments comprised encoding and recognitions runs. In the encoding runs, the participants were asked to observe presented visual stimuli with eye movements allowed. In the recognition runs, the participants were asked to observe presented visual stimuli with eye movements allowed and to answer whether each image had been presented in the preceding encoding runs. The encoding and recognition runs were combined and performed in the order presented in the figure, preceded by calibration of the eye tracker. This sequence was performed five times for each participant.

The eye movement of the participants was measured using an infrared camera system at a sampling rate of 1000 Hz, which is fast enough to capture the participants’ fixation location. The eye movement was recorded for both the encoding and recognition runs (Fig. 1), but only results for the encoding runs are presented in this section and the recognition runs were not analyzed because half of the visual stimuli in the recognition runs had been presented in the encoding runs, which might affect perceptual states.

### Gaze attraction of visual features

For each visual stimulus, we generated the spatial distribution of visual features using AlexNet^15^ pretrained with the ImageNet database^21^ to classify images into 1000 object categories and the SmoothGrad technique^22^.

AlexNet is a DCNN model consisting of five convolutional layers and three fully-connected layers, and hierarchical visual features are extracted through its layered architecture. For example, simple visual features like grating patterns are represented in layer 1, and more complex features like texture- or object-like patterns are represented in the higher layers of the model. It is one of the most accepted DCNN models for the hierarchical human visual system because of its similarity in the architecture and activation characteristics^16–18^, particularly regarding with the hierarchical correspondence between the brain and DCNN models based on bidirectional (DCNN-to-brain and brain-to-DCNN) predictability^23^ (see also another evaluation by Schrimpf et al.^24^ based on predictability of neural activity from DCNN model).

**Figure 2:**
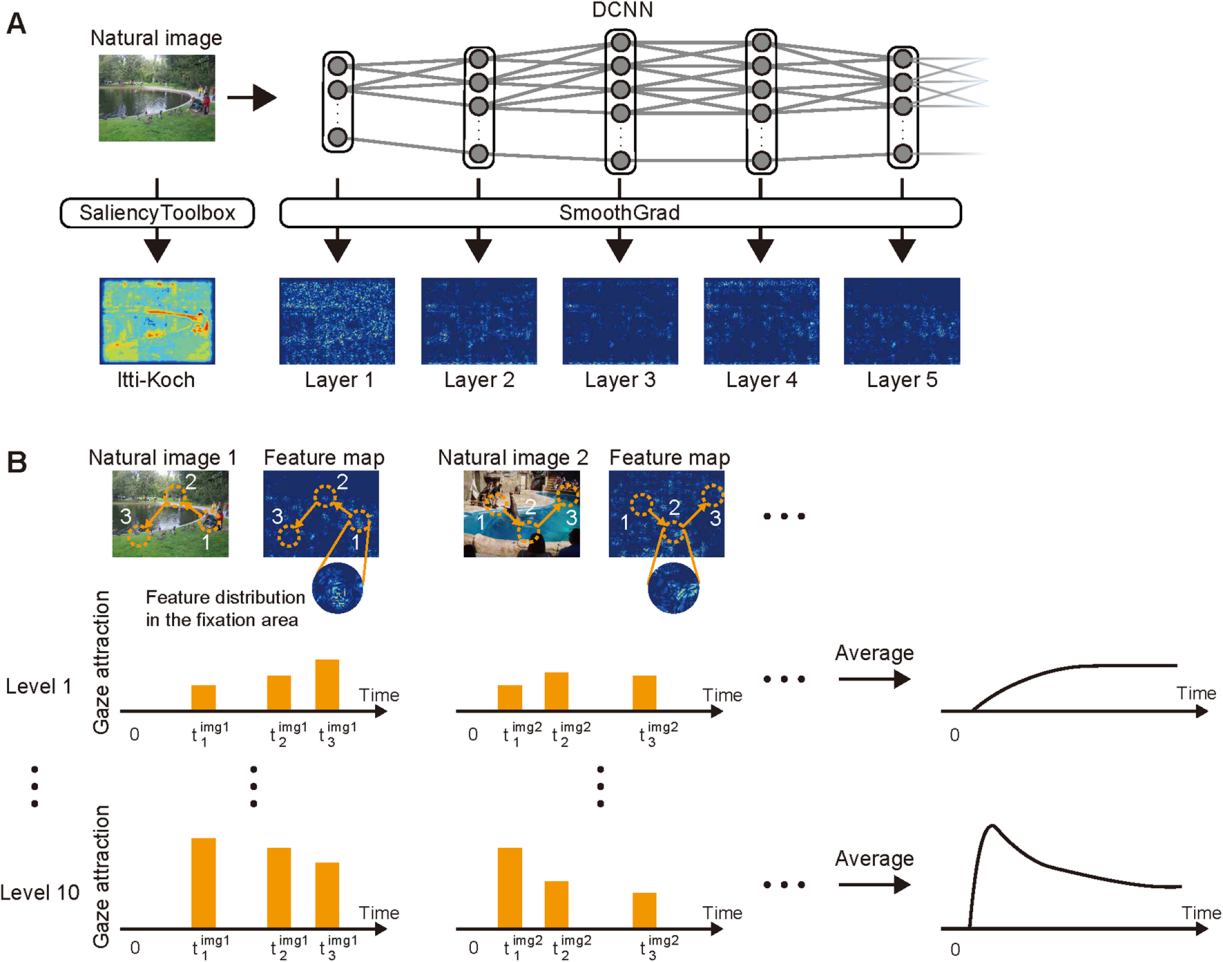
Feature maps and gaze attraction. (**A**) Feature maps corresponding to hierarchical layers of the DCNN and the Itti–Koch saliency. The unit activation in each layer of the DCNN model is back-projected to the image-pixel space using the SmoothGrad technique for each visual stimulus. The feature intensity is represented by pseudocolor (blue, low; red, high). The intensity of the Itti–Koch saliency is also represented similarly. (**B**) Time course of the gaze attraction of visual features. The combination of fixation data and the feature map provide how much of a visual feature is contained in each fixation area for a specified feature intensity level. This procedure defines the gaze attraction of each visual feature for the intensity level at the time when the fixation occurs. The average of these values over all visual stimuli then produces the time course of the gaze attraction of each visual feature for each intensity level.

SmoothGrad is a method to back-project activation values in a layer of a DCNN model into the pixel space of the input image to make a graded map representing which locations have high/low values corresponding to the activation of the specified layer. Since if particular locations in the presented image have visual features that can activate a particular layer in the DCNN model, the locations can be considered to have “intense” visual features for the corresponding layer. Thus we can interpret that the graded value represents “feature intensity,” and its map is called a “feature map” (Fig. 2A). Here we focused on only the five convolutional layers as the target to apply SmoothGrad to analyze the visual features. In addition, we also calculated the Itti–Koch saliency map (Fig. 2A) as a typical example of the lower-order visual feature conventionally used in human gaze studies. Because the intensity of visual features is presumed to affect the fixation, we discretized the feature intensity by evenly dividing them into ten levels between the minimum to the maximum value for each entire stimulus and treated feature maps at the different intensity levels separately.

Combining the above experimental datasets of eye movement recording and the feature maps for each presented stimulus, we defined an index representing how much a particular feature exists in the fixated area (defined by the 1-deg circular region around the fixated point) by calculating the ratio of the area occupied by the corresponding feature in the fixated area (Fig. 2B, see “Methods”). We can interpret a high value of this index as a consequence of the gaze being attracted to the location by the visual feature existing there. We thus call this index “gaze attraction of the visual feature” (Fig. 2B).

Since the eye movement data consisted of a sequence of multiple fixations over time, we quantified the gaze attraction of a particular visual feature at a particular intensity level for every fixation for each presented stimulus. Then taking the average over all presented stimuli, we can illustrate the time course of the gaze attraction of the visual feature at each intensity level (Fig. 2B).

### Time course of gaze attraction

**Figure 3:**
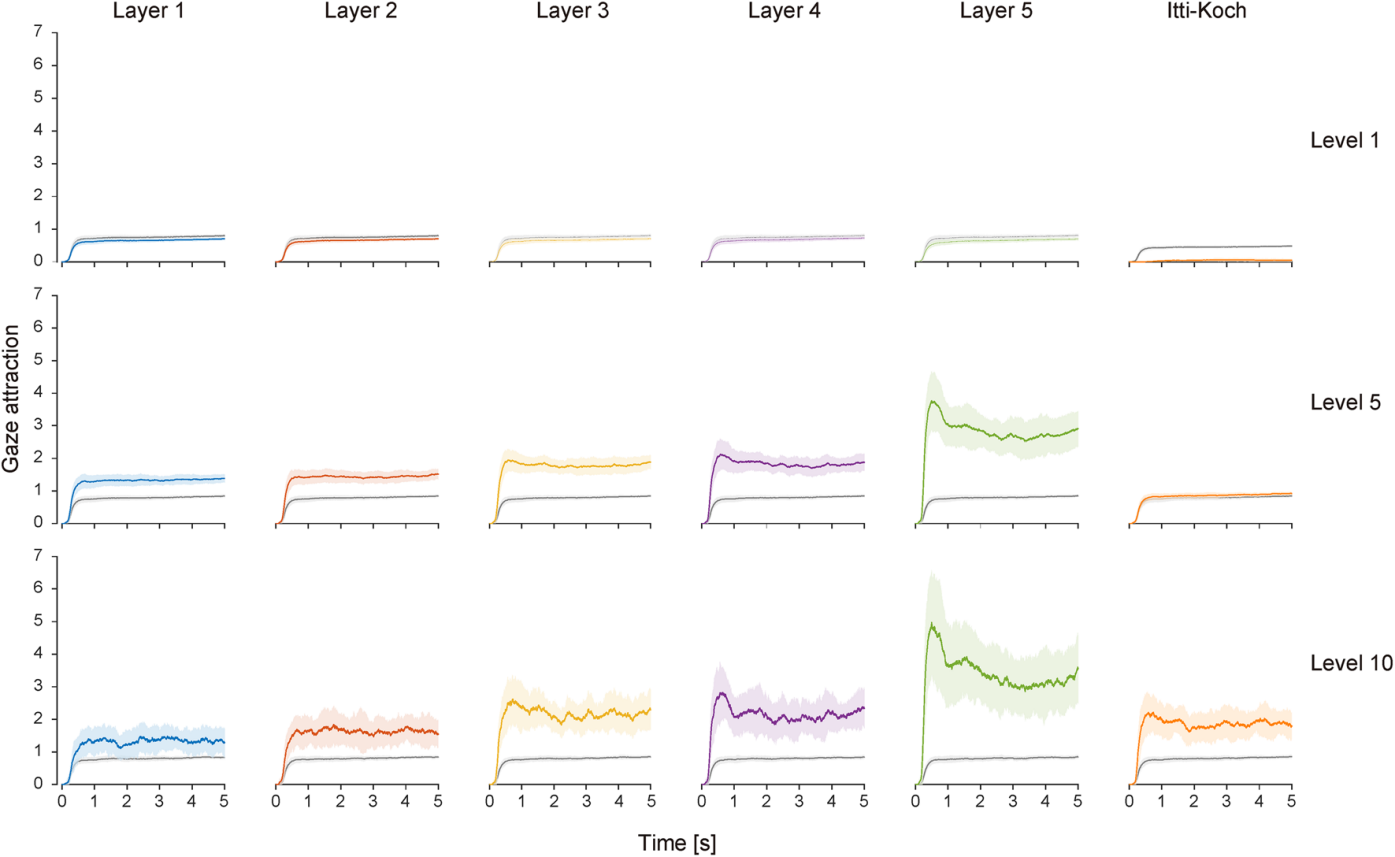
Time courses of gaze attraction of visual features. Three levels of feature intensity (top row, level 1; middle row, level 5; bottom row, level 10) are shown as representative examples. Results from each visual feature are aligned in the column direction. In each graph, the colored solid line and shading indicate the mean and standard deviation over participants, and the gray solid line and shading indicate the mean and standard deviation of the chance level over participants, respectively. The time origin indicates the onset of visual stimulus presentation.

Figure 3 shows the representative examples of the time courses of the gaze attraction of the visual features for the three intensity levels of 1 (lowest), 5 (middle), and 10 (highest), compared with the chance level calculated from the randomized fixation locations (see “Methods”). For the visual features at low-level intensity (top row in Fig. 3), the gaze attraction was almost at the same level as chance (Layers 1–5) or lower (the Itti–Koch saliency). There was no major difference between visual features except the Itti–Koch saliency because all feature intensities were low and their characteristics were thus lost. The difference from the chance level became evident as the intensity increased (middle to bottom rows in Fig. 3), indicating that the gaze was attracted to locations with intense visual features. The difference in the gaze attraction between the visual features also became prominent for the higher intensity levels (middle to bottom rows in Fig. 3).

### Spatial gaze bias

**Figure 4:**
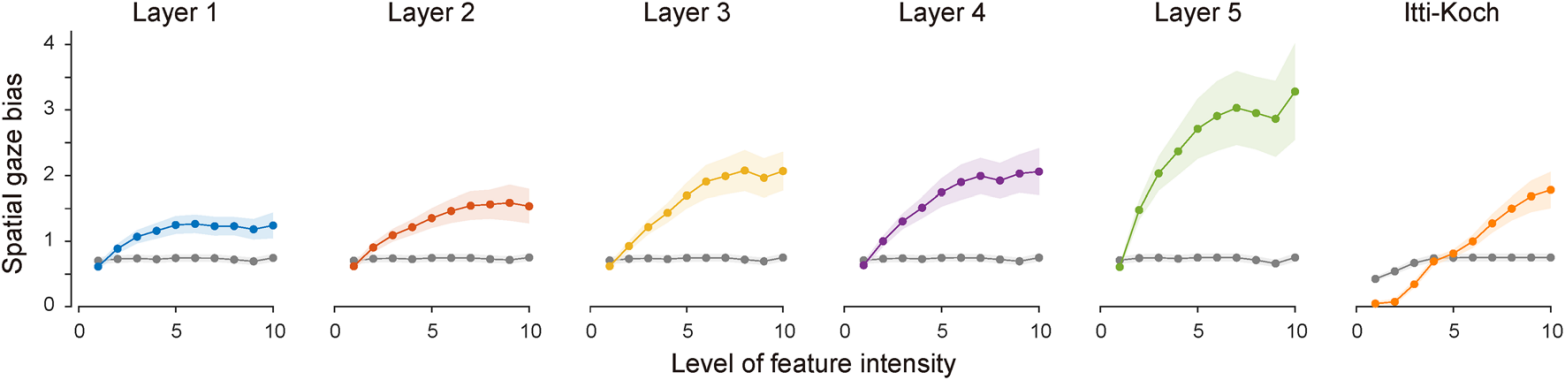
Spatial gaze bias for visual features. The spatial gaze bias for each visual feature is plotted against the feature intensity level. In each graph, the colored solid line and shading indicate the mean and standard deviation over participants, and the gray solid line and shading indicate the mean and standard deviation of the chance level over participants, respectively.

By taking the mean of the gaze attraction of the visual features over time, we can simply ignore the temporal evolution and quantify an index of how the gaze was spatially biased toward certain features. We call this index “spatial gaze bias” (see also “Methods”). The spatial gaze bias progressively increased with the hierarchy of the convolutional layers (Fig. 4; two-way ANOVA, Bonferroni-corrected p < 0.05 for multiple comparisons; the spatial gaze bias increased in the order of Itti–Koch, Layer 1, 2, 3, 4, and 5 (pair-wise significance was not found between Layer 3 and 4; all other pair-wise comparisons were significant), indicating that the higher-order visual features activating higher layers of the DCNN model were stronger gaze attractors than the lower-order visual features activating lower layers of the DCNN model. These results are consistent with previous studies showing higher-order features outperforming conventional saliency in the prediction of the fixation map^8^.

An unexpected result was found for the Itti–Koch saliency, showing locations with low feature intensity were seen less than the chance level. We assumed that this result was not due to such locations being avoided but due to the center bias of fixations^25^ and the artifactual outcomes of the Itti–Koch saliency computation that output low intensity near the edge of visual stimuli (Supplementary Fig. 3). More details are described in “Discussion” section.

### Temporal gaze bias

**Figure 5:**
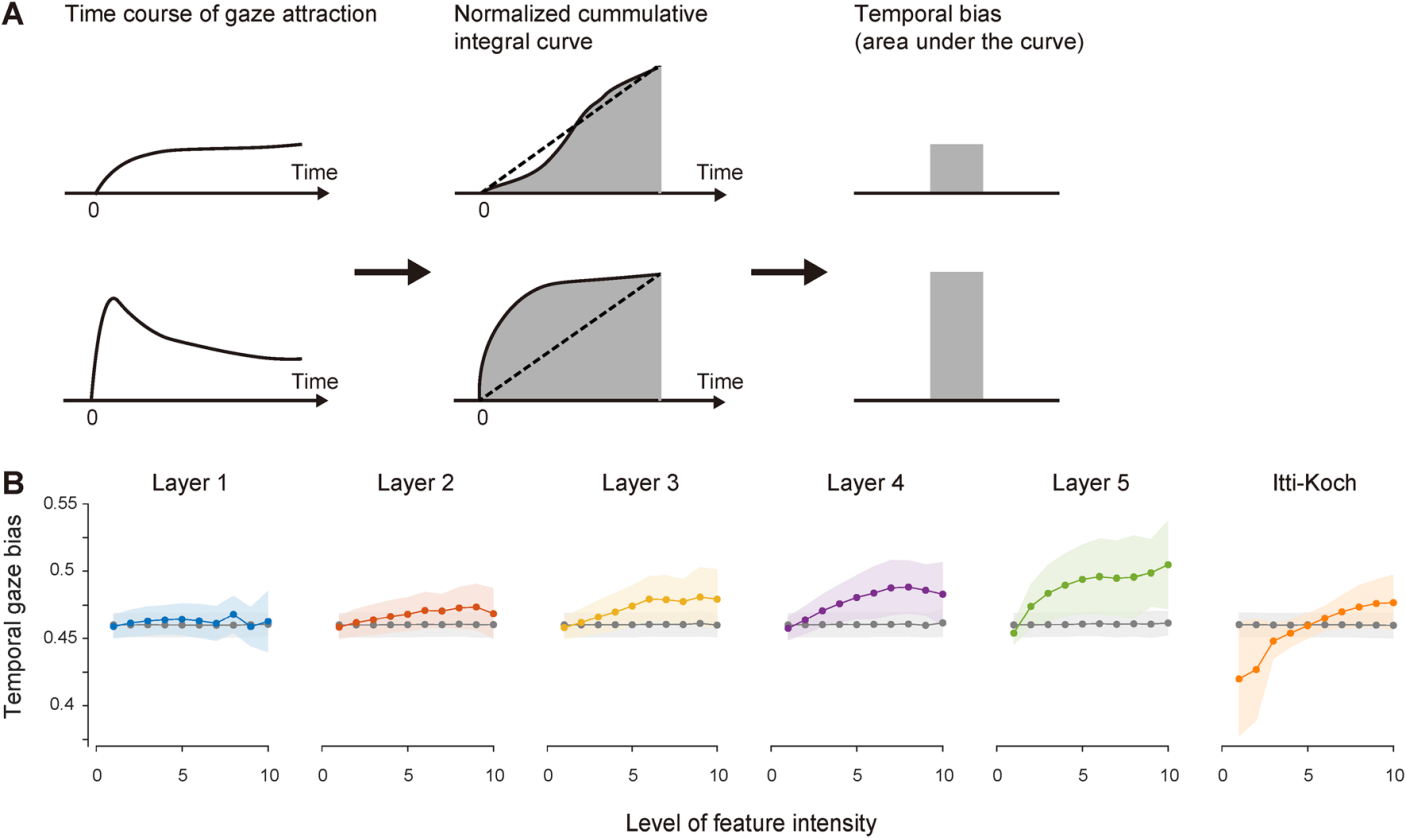
Temporal gaze bias for visual features. (**A**) Procedure in quantifying the temporal gaze bias from the time course of the gaze attraction. The time course of the gaze attraction is integrated cumulatively and then normalized by the maximum value. The convexity of the normalized cumulative integral curve indicates how fast the time course of the gaze attraction reaches a peak value, and the area under the curve serves as a measure of the temporal gaze bias. (**B**) Temporal gaze bias calculated in this procedure. Results are plotted against the feature intensity level. In each graph, the colored solid line and shading indicate the mean and standard deviation over participants, and the gray solid line and shading indicate the mean and standard deviation of the chance level over participants, respectively.

The difference between visual features was also evident in the temporal characteristics of the gaze attraction (here, the “temporal” characteristics mean the development of the gaze attraction over time). The time course of the gaze attraction of the visual feature was not flat over time but peaked after the stimulus presentation (at approximately 500 ms), particularly for the visual features corresponding to the higher layers (middle to bottom rows in Fig. 3) at higher feature intensity. To quantify this temporal inhomogeneity, we defined the temporal gaze bias, which is a measure of how fast the gaze is attracted to the visual feature (Fig. 5A; see details in “Methods”). Figure 5B shows that the temporal gaze bias was greater for the higher-order visual features activating higher layers of the DCNN model than the lower-order visual features activating lower layers of the DCNN model and the Itti–Koch saliency, particularly at the higher level of feature intensity. Statistical analyses showed that the temporal gaze bias increased in the following order: the Itti–Koch saliency, Layer 1, 2, 3, 4, and 5 (two-way ANOVA, Bonferroni-corrected p < 0.05 for multiple comparisons; pair-wise significance was not found between the Itti–Koch saliency vs. Layer 1, Layer 1 vs. 2, Layer 2 vs. 3, Layer 3 vs. 4; all other pair-wise comparisons were significant). The temporal gaze bias increased as a function of feature intensity, except in the lower layers such as Layer 1 and 2 (p < 0.05 for interaction between visual features and feature intensity, two-way ANOVA), indicating that the gaze is attracted to the spatial locations with visual features that strongly activate the higher layers of the DCNN model, particularly during the initial period after the stimulus onset.

Note that a temporal gaze bias smaller than the chance level was also found for the Itti–Koch saliency for the same reason described in the previous section (see also the Discussion section).

### Latency of gaze bias

**Figure 6:**
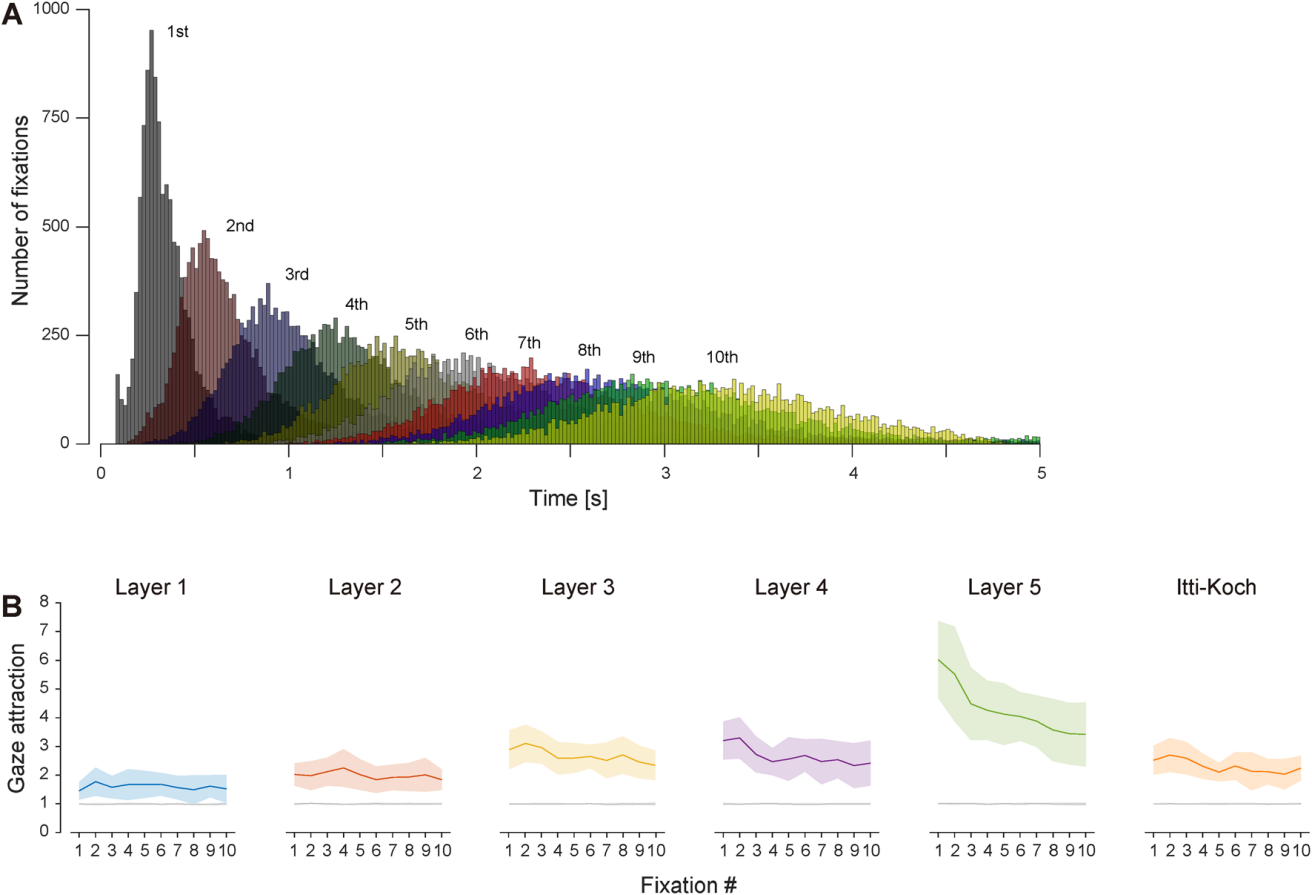
Latency of fixations. (**A**) Latency distribution of fixations sorted by its order (up to the 10th fixation; bin width, 20 ms). (**B**) Gaze attraction of visual features sorted by the fixation order. Only results for feature intensity at level 10 are shown here. In each graph, the colored solid line and shading indicate the mean and standard deviation over participants, and the gray solid line and shading indicate the mean and standard deviation of the chance level over participants, respectively.

To examine how fast the effect of the gaze attraction of the visual features emerges, we evaluated the latency (the onset time of the fixation relative to stimulus onset) of each fixation (up to the 10th fixation) and what visual features attracted the gaze at each fixation order. The first fixation had peak latency at the 260–280 ms bin, which is similar to the result of a previous study using natural scenes as stimuli^13^, though a much faster gaze was observed in the distribution. Another small peak was found for the first fixation latency at the 80–100 ms bin, but this would be the anticipatory component independent of the visual stimulus observation that remains even after removing such eye movements (see “Methods”). We then examined what visual feature attracts the first fixation by comparing the gaze attraction of each visual feature. Figure 6B shows that the gaze attraction at the first fixation was significantly higher for the visual feature corresponding to Layer 5 than other features (one-way ANOVA for the gaze attraction at the first fixation, Bonferroni-corrected p < 0.05 for multiple comparisons). Figure 6B only shows results at the largest feature intensity level (level 10) as representative examples, but the significance of the Layer 5 feature to attract the first fixation was found at level 2 and above (one-way ANOVA for the gaze attraction at the first fixation, Bonferroni-corrected p < 0.05 for multiple comparisons). These results indicate that the higher-order features attract the human gaze strongly even at the first fixation, and such biases can emerge earlier than 260–280 ms.

## Discussion

In this study, we examined what and when visual features are fixated on by measuring eye movements and analyzing visual features embedded in the presented visual stimulus. To reveal the direct relationship between the human gaze and visual features, we used a DCNN model by which feature maps can be identified for each visual feature along the DCNN model hierarchy from the lower layer (Layer 1) to the higher layer (Layer 5). We also used the Itti–Koch saliency map for the purpose of comparison as the most conventional visual feature that has been extensively studied in human gaze studies. Using the measured fixation data and the feature maps, we quantified the gaze attraction of the visual features by how much they were contained in the fixated area. In contrast with the previous model, our approach did not combine any read-out module with the DCNN model to predict fixation locations but analyzed visual features at the fixation locations, allowing us to clarify the direct contribution of each visual feature to attract the fixations.

As a result, we revealed that higher-order visual features corresponding to the higher layers were a stronger gaze attractor than lower-order visual features corresponding to the lower layers and the Itti–Koch saliency. The spatial locations containing much higher-order visual features were fixated prominently in the early period after visual stimulus presentation, even at the first fixation (Fig. 6B), and the preference for the higher-order visual features continued during observation of the visual stimulus (Fig. 3). Analyses further showed that this tendency strengthened with the feature intensity (see “Results” and “Methods” for its definition), suggesting that the higher-order visual features contribute to attracting the fixations. This is clear experimental evidence of the importance of the higher-order visual features over the lower-order visual features and the conventional saliency to explain the human gaze bias during the observation of natural scenes.

According to these results, one might assume that the fixation locations could be better predicted from activation in the higher layer of the DCNN model than that in the lower layer. Since the purpose of this study was to see the relationship between the human gaze and hierarchical visual features, we did not perform fixation prediction using activation in each layer in the DCNN model. However, as previous studies^8,13^ partially showed that the higher layer activation in the DCNN model is useful for accurate prediction of the fixation locations, it is highly likely that the prediction accuracy for the fixation locations would be higher if using activation in the higher layers than in the lower layers in the DCNN model we currently used.

What brain areas play an important role in extracting these higher-order visual features to attract the human gaze? AlexNet, the DCNN model we used in this study, was developed for the image-based object recognition task, and its architecture is designed to resemble hierarchical visual systems in the brain, particularly along the ventral pathway. Previous studies showed that neural activity in the higher visual cortex in the ventral pathway of the monkey brain can be predicted from unit activation patterns in the higher layer of AlexNet^18^. Human imaging studies also showed that functional magnetic resonance imaging signals of the higher visual cortex in the ventral pathway can be predicted from unit activation patterns in the higher layers of the DCNN model^16^ and vice versa^17^. These studies indicate a correspondence between the hierarchical structures of the brain and those of the DCNN model. This homologous relationship suggests that the higher-order visual features are extracted in the higher visual areas in the ventral pathway and contribute to attracting the human gaze. The strong gaze attraction of a higher-order feature was observed even at the first fixation (Fig. 6B), whose peak latency was approximately 260–280 ms. The exact identification of the shortest latency of the first fixation is difficult because of the contamination of anticipatory components, but a reasonable estimate could not be earlier than 120 ms corresponding to the trough of the latency distribution of the first fixation and also roughly corresponding to the shortest end of the second fixation latency distribution (Fig. 6A). If we adopt this value as the shortest latency of the gaze bias to the higher-order visual feature, it is still slower than the putative activity latency of the higher visual areas of the human brain^26^. It is thus consistent in terms of behavioral and neural latency if the feature extraction in the higher visual areas is the origin of the gaze bias.

The conventional saliency defined by the Itti–Koch model^2^ has been considered a good fixation predictor, particularly in the early period after the stimulus presentation^11,12^. Such a tendency was actually observed in our data. When the feature intensity was high, its gaze attraction was greater than chance during the period of visual stimulus presentation, with a peak in the early period after the stimulus onset [the transient peak was detected at 580 ms for level 8, 575 ms for level 9, and 592 ms level 10, respectively, within the period during which the gaze attraction showed statistically higher values (t-test, p < 0.05) than the steady state (the last 1-s period of each trial)], showing that the temporal gaze bias was larger than chance when the feature intensity was larger than level 8. However, compared with the higher-order visual features, the gaze attraction of the Itti–Koch saliency was limited. The gaze attraction of the higher-order visual features was overwhelmingly high in the early period after the visual stimulus presentation, and it continued at a certain level beyond chance until the end of the visual stimulus presentation (Fig. 3). These results are consistent with those of a previous study^13^ analyzing the relationship between visual features activating higher layers in a DCNN model and eye movements, though their model was indirect to clarify the contribution of visual features to the gaze bias, suggesting the explanatory power of the higher-order visual features for the spatiotemporal gaze bias.

Another concern about the Itti–Koch saliency^2^ is its multiscale nature based on a Gaussian pyramid^27^, which may complicate the discussion on brain areas involved in its computation. Our results show that the gaze attraction of the Itti–Koch saliency was approximately between Layers 2 and 3 at its highest intensity level (Fig. 3), although the saliency computation was based on visual features for which the early visual area has a preference^28^. There could be a discrepancy between brain areas that are supposed to be necessary for the feature preference and the feature extraction scale.

Among the visual features tested in this study, only the Itti–Koch saliency had a gaze bias lower than the chance level when the feature intensity was low. This result could be interpreted as if the gaze was less directed at the locations of low intensity for the Itti–Koch saliency. However, it is highly likely that the result would be an artifact due to the procedure to compute the Itti–Koch saliency near the edge of the presented stimuli. The Itti–Koch saliency defined by their model for the particular locations requires its surrounding pixel values, but the pixel values cannot be fully provided near the edge of the presented stimuli since a part of them are outside of the stimuli where the pixel values are undefined. Consequently, the intensity of the Itti–Koch saliency should be low near the edge of the presented stimuli. In addition, the human gaze was more directed around the center of the presented stimuli (center bias effect^25^). Therefore, the result looks as if the gaze would be less directed to the locations with lower intensity of the Itti–Koch saliency, which are actually distributed near the edge of the presented stimuli, than the chance level that was computed by random assignment of coordinates to the fixated locations with uniform probability across the whole presented stimuli, irrespectively of the center bias effect.

We used AlexNet, one of the most typical DCNN models, to define hierarchical visual features. This procedure was based on the assumption that AlexNet is a proxy of the human hierarchical visual systems. Although many studies suggest the similarity between them, there should also be differences that might affect the results observed in this study. For example, the human brain has feedback connections from multiple areas, but AlexNet does not. It plays an important role in visual information processing and perhaps in gaze control as well. The difference in model architecture, training procedure, and stimuli to evaluate the layer activation could also influence the results^29–31^. It might be interesting issues in future work to address how such differences influence the human gaze.

In this study, we found that the higher-order visual features are strong gaze attractors, particularly immediately after the onset of the visual stimulus presentation. This evidence suggests the possibility that the human gaze could be guided to specified locations by incepting higher-order visual features with sufficient intensity. Such techniques could be adopted in a critical test of the current findings. The artificial manipulation of visual features could also be useful in designing traffic signs, fail-free user interfaces, and effective advertisements. Thus, our approach could provide a new method of exploring and even utilizing the relationship between visual features and induced eye movement.

## Methods

### Participants

Twenty participants [16 males, 4 females; age ranging between 20 and 26 years (mean, 21.7 years)] participated in the experiment. All participants had normal or corrected-to-normal vision acuity. They were compensated by 1000 JPY per hour for their participation. All participants gave written informed consent before participating in the experiments. The procedure was approved by the institutional review board of the University of Electro-Communications. All methods were performed in accordance with the relevant guidelines and regulations.

### Visual stimuli

Images of natural scenes were selected from the ADE20K dataset^20^ and the PASCAL-Context dataset^19^, in which objects are segmented separately and annotated with corresponding object categories. We used color images of more than four object categories with an aspect ratio of 4 (horizontal) vs. 3 (vertical) as visual stimuli for the experiment. The number of selected images was 496 from the ADE20K dataset and 154 from the PASCAL-Context dataset. Each image was rescaled to 800 × 600 pixels (width × height).

### Apparatus

Visual stimuli were presented on a 21-inch CRT monitor [FlexScan T966, EIZO NANAO Inc.; frame rate, 60 Hz; resolution, 1024 × 768 pixels (width × height)]. Participants were seated at a distance of 86 cm from the monitor, and the visual stimuli subtended a field of view of approximately 20° × 15° (width × height). A chin rest was used to keep the participant’s head still. During the visual stimulus presentation, the participant’s right eye movements were recorded with EyeLink 1000 (SR Research Ltd.) desktop mount system at a sampling rate of 1000 Hz. An image of a small white square was displayed synchronously with the visual stimuli at the left side of the CRT monitor, and a light sensor was attached on the monitor to detect the onset of visual stimulus presentation. The detected onset was used to define the time origin for the recorded eye movement precisely. The light sensor was covered with a dark cloth so that participants saw neither the white square nor the light sensor during the experiment. Visual stimulus presentation was controlled by MATLAB (The MathWorks, Natick, MA) using Psychophysics Toolbox Version 3^32–34^).

### Experimental design

The experiments comprised encoding and recognition runs. In the encoding runs, the participants were asked to observe presented visual stimuli with eye movements allowed. In the recognition runs, the participants were asked to observe presented visual stimuli with eye movements allowed and to answer whether each stimulus had been presented in the preceding encoding runs.

After the eye tracker was calibrated using a nine-point fixation presented on the monitor, three sets of the combination of an encoding run and a recognition run were performed (Fig. 1). There were 40, 39, and 39 stimulus presentation trials in the encoding runs, each of which was followed by eight stimulus presentation trials in the recognition runs. This sequence (calibration and the three sets of the combination of the encoding and recognition runs) was repeated five times, resulting in 590 (450 from the ADE20K dataset and 140 from the PASCAL-Context dataset) and 120 (92 from the ADE20K dataset and 28 from PASCAL-Context dataset, with half being the same images as those in the encoding runs for the recognition task) natural scene presentations for encoding and recognition, respectively. The eye tracker was recalibrated between runs when necessary.

In the encoding runs, the participants pressed the space key on a keyboard to start each trial. A white fixation cross (0.3° × 0.3°) was then presented for 500 ms at the center of the CRT monitor on a black background, and an image of a natural scene was subsequently presented as a visual stimulus. The participant was instructed to observe the presented stimulus with free eye movements while keeping their head still on the chin rest. The presented stimulus automatically disappeared at 5000 ms after the onset of the stimulus presentation, and the monitor turned black until the participant pressed the space key to start the next trial (the instruction to press a button appeared on the screen; see Fig. 1A). The participant’s right eye movements were continuously monitored during the trial. The eye tracker automatically recognized saccade events if the speed of eye movement exceeded 30°/s, and the remaining events were recognized as fixation events after removing blinks. The spatial coordinates and event time of each fixation were recorded by the eye tracker. Fixation events that happened 80 ms after stimulus presentation were considered anticipatory and excluded from the analysis^35^.

In the recognition runs, the participants again pressed the space key on a keyboard to start each trial. A white fixation cross (0.3° × 0.3°) was presented for 500 ms at the center of the CRT monitor on a black background, and an image of a natural scene that was either one presented in the preceding encoding run or one new to the experiment was then presented as a visual stimulus. There were four previously presented stimuli in each recognition run; i.e., the probability of seeing a previously presented image was 50%. The participant was instructed to observe the visual stimuli with free eye movements while keeping their head still on the chin rest. The presented stimuli automatically disappeared at 2000 ms after the onset of the stimulus presentation and the monitor turned black. The participant was instructed to press the “z” key on the keyboard during this period if they thought the stimulus had been presented in the preceding encoding run or press the slash key if not, without a time constraint (the instruction to press a button appeared on the screen; see Fig. 1A). After answering the task, the participants pressed the space key to start the next trial.

### Visual feature analysis

#### DCNN feature map

DCNN feature maps for each stimulus were computed using AlexNet (Fig. 2A) pretrained with the ImageNet database^21^ to classify images into 1000 object categories. AlexNet comprised five convolutional layers and three fully-connected layers. Each of the five convolutional layers consisted of convolutional image filtering followed by nonlinear rectification, maximum value selection within the spatial window (max pooling), and normalization. The three fully-connected layers were not considered as the target of analysis because we were interested in preserving spatial information of extracted visual features as much as possible, as in the previous studies^7,8,13^.

The feature maps were then generated from the five convolutional layers using SmoothGrad^22^. SmoothGrad is a method originally used with DCNN models for object classification. DCNN-based object classification is typically done by comparing activation values of all neural network units at the final layer, and each unit corresponds to each object category. SmoothGrad can back-project the activation of each unit in the final layer into the pixel space of the input image, allowing visualization of the areas that are important for the classification of the target object. However, it can be generally used to identify spatial locations in an image important to yield activation in any layers of DCNN models by back-projecting the activation into the pixel space of the image. In this paper, we utilized this function of SmoothGrad and applied it to each layer’s activation induced by stimuli presented to participants in our experiments, generating maps that represent the contribution of each spatial location in the stimulus to the activation in each layer. The generated maps have a graded value, which indicates the difference in contribution to evoke activation in each layer. In the case of AlexNet, since each layer is known to be activated by hierarchical visual features, such as lower-order visual features activating layer 1 and higher-order visual features activating layer 5^15^, pixel values back-projected from the activation in each layer by SmoothGrad can be considered to represent the spatial distribution of visual features that activate the corresponding layer. In this sense, we may interpret the graded value as “feature intensity” because a spatial location with a higher value for a particular feature is expected to yield larger activation in the corresponding layer. Thus we call the map made by SmoothGrad a “feature map”. Since our purpose is to visualize the spatial distributions of visual features corresponding to each layer, not to a particular single unit in the layer, of the DCNN model, activation values of all units in each layer were back-projected into the pixel space of the presented stimulus to generate the feature map. This procedure was repeated for all stimuli and for all five convolutional layers of the DCNN model, resulting in five feature maps for each stimulus (Fig. 2A).

#### Itti–Koch saliency map

The Itti–Koch saliency map for each stimulus was computed using SaliencyToolbox^3^, a conventional saliency model that combines conspicuity maps of luminance contrast, color contrast, and orientation with equal weights. The Itti–Koch saliency map shows a distribution of the intensity of the feature, similar to the feature maps defined by the DCNN model and SmoothGrad (Fig. 2A).

#### Quantitative evaluation of gaze attraction of visual features

We defined the gaze attraction of a particular visual feature as how much of the visual feature was contained in the fixated location (Fig. 2B). Here the visual feature indicates a pixel pattern in the region that activates either layer 1–5 of the DCNN model (AlexNet). In addition, we also used the Itti–Koch saliency as a visual feature. To consider the effect of the intensity difference in visual features (the definition of “intensity” was defined earlier), we first rescaled the feature intensity into the range between 0 (minimum) and 1 (maximum) for each stimulus and discretized it into ten levels. Here we treated the visual features of different intensities as different features (for example, visual features corresponding to Layer 1 of level 1 and Layer 1 of level 2 were treated separately).

Given the feature maps labeled with the feature intensity levels, we could identify what visual features were dominant at the fixated point and thus derive a quantity for the gaze attraction. However, this procedure might be inaccurate because the fixation locations identified by the eye tracker contain measurement errors, which are expected to be within 1° of the visual angle according to the specifications of the eye tracker. In addition, since the human fovea is about 2° in its diameter^36^, it would be reasonable to assume that the target visual information is collected during fixation at this spatial resolution. Therefore, instead of using a single point in the stimulus, we defined “fixation area” as a circular region of 1° radius around each fixated location and calculated how much of the fixation area was occupied by each visual feature in the region. If there is a difference in the total area occupied by each visual feature in the entire image, it yields spurious biases in the occupancy rate of each visual feature in the fixation area since it was calculated by counting the number of pixels in the fixation area and divided by the total number of pixels in the fixation area. To avoid such effects, we divided the occupancy rate of each visual feature in the fixation area by the total occupancy rate of each visual feature in the entire image (the number of pixels for the corresponding visual feature in the entire image divided by the total number of pixels in the entire image). This relative occupancy rate thus indicates how dominant each visual feature is in the fixation area, which we defined as the gaze attraction of the visual feature.

This procedure can be described in the mathematical expression as follows. Suppose there is the visual feature *F* that has the feature intensity level *L* in the image ${I}_{n}$, the gaze attraction of the visual feature at time *t* after the onset of the image presentation, $GA\left({F}_{L},t,{I}_{n}\right)$, can be expressed as

$$GA\left({F}_{L},t,{I}_{n}\right)=\left\{\begin{array}{cc}\frac{{N}_{{F}_{L}}^{FA}}{{N}_{all}^{FA}}/\frac{{N}_{{F}_{L}}^{{I}_{n}}}{{N}_{all}^{{I}_{n}}}& \text{if }t \text{is the time when a fixation event is recognized}\\ & \\ 0& {\text{otherwise}}\end{array}\right.,$$

where ${N}_{{F}_{L}}^{FA}$ is the number of pixels corresponding to the visual feature *F* at the feature intensity level *L* in the fixation area (*FA*), ${N}_{all}^{FA}$ is the total number of pixels in the fixation area, ${N}_{{F}_{L}}^{{I}_{n}}$ is the number of pixels corresponding to the visual feature *F* at the feature intensity level *L* in the entire area of the image ${I}_{n}$, and ${N}_{all}^{{I}_{n}}$ is the total number of pixels in the entire area of the image ${I}_{n}$, respectively. Here the numerator, $\frac{{N}_{{F}_{L}}^{FA}}{{N}_{all}^{FA}}$, corresponds to the occupancy rate of the visual feature *F* at the intensity level *L* in the fixation area and the denominator, $\frac{{N}_{{F}_{L}}^{{I}_{n}}}{{N}_{all}^{{I}_{n}}}$, corresponds to the occupancy rate of the same visual feature in the entire image. Then by taking an average over all presented images ${I}_{n}$ (*n* = 1…${N}_{I}$; ${N}_{I}$, number of images), we get the time course of the gaze attraction of the visual feature *F* at the intensity level *L* as

$$GA\left({F}_{L},t\right)=\frac{1}{{N}_{I}}\sum_{n=1}^{{N}_{I}}GA\left({F}_{L},t,{I}_{n}\right).$$

Since this is the time course from a single participant, we further took an average over participants and obtained the mean time course of the gaze attraction of the visual feature at different intensity levels (Fig. 3).

#### Spatial gaze bias

To show how often the fixation was directed to each location in the presented stimuli, conventional studies, whether experimental or computational approaches, have computed fixation maps. The fixation map is typically defined by counting all fixations during the stimulus presentation while ignoring when the fixation happens after the stimulus onset. Similarly, to evaluate how frequently the gaze was attracted to spatial locations containing each visual feature with a specified intensity level, we simply took the average of the time course of the gaze attraction of each visual feature at the intensity level. We defined this quantity as the spatial gaze bias to the visual feature.

#### Temporal gaze bias

The gaze attraction of the visual feature is not constant but changes dynamically over time. As shown in Fig. 3, some visual features had a clear peak in the early period after the visual stimulus presentation and others did not. To quantify such a difference in temporal characteristics, we took a cumulative integral over the time course of the gaze attraction (Fig. 5A). The time course of the gaze attraction comprises non-negative values, and its cumulative integral thus becomes convex if the time course shows higher values close to the stimulus onset whereas it becomes concave if the time course shows the opposite tendency. Thus, the convexity of the cumulative integral of the time course of the gaze attraction reflects how quickly the visual feature is fixated after the stimulus onset. We here quantified the convexity using the area under the cumulative integral of the time course of the gaze attraction. We defined this quantity as the temporal gaze bias to the visual features. Note that the time course of the gaze attraction was normalized such that its minimum-to-maximum range becomes 0 to 1 before computing the temporal gaze bias because the constant component of the time course affects the convexity of the cumulative integral although it is irrelevant to the temporal gaze bias. The cumulative integral was also normalized such that its minimum-to-maximum range became 0 to 1 so as to evaluate its convexity independently of the absolute values of the cumulative integral.

We confirmed that this procedure faithfully measures the temporal gaze bias by simulation analysis using hypothetical time courses of the gaze attraction (see Supplementary information). The temporal gaze bias varied consistently with model parameters that control the peak time (Supplementary Fig. 1A,B) and the magnitude of the transient component (Supplementary Fig. 1C,D) of the time course of the gaze attraction, as expected.

#### Chance levels of spatial and temporal gaze bias

It is difficult to define the chance levels of the spatial and temporal gaze bias theoretically, and we thus adopted the following empirical procedure to define them. For each participant, fixation coordinates measured for all 590 trials in the encoding runs were discarded and random coordinates uniformly sampled from the entire stimulus dimension were reassigned for all fixation data while their timing information remained unchanged. The time course of the gaze attraction was then computed in the same way as for the original fixation data. This operation was conducted 100 times and the time courses were averaged. The averaged time course of the gaze attraction with randomized coordinates keeps the mean frequency of the fixation at each timing (because the timing information is preserved), but the occupancy rate of each visual feature in a reassigned fixation location changes and asymptotically approaches the mean density of each visual feature in the entire stimulus, serving as the time course of the chance-level gaze attraction of each visual feature for each participant. The chance level for the spatial and temporal gaze bias was calculated from the time course of the chance-level gaze attraction using the same procedure explained above.

## Supplementary Information

Supplementary Information.

## Data Availability

The datasets generated and analyzed during the current study are available from the corresponding author on reasonable request.
